# Augmenting Bragg Reflection with Polymer-sustained Conical Helix

**DOI:** 10.1038/s41598-019-41836-4

**Published:** 2019-04-02

**Authors:** Vinay Joshi, Daniel A. Paterson, John M. D. Storey, Corrie T. Imrie, Liang-Chy Chien

**Affiliations:** 1 Chemical Physics Interdisciplinary Program and Advanced Materials & Liquid Crystal Institute, Kent State University, Kent, 44242 USA; 2 Department of Chemistry, School of Natural and Computing Sciences, University of Aberdeen, Aberdeen, AB24 3UE Scotland UK

## Abstract

There has been a recent surge of interest in smart materials and devices with stimuli-responsive properties for optical modulations. Cholesteric liquid crystals (CLCs) are a unique class of light-manipulating materials, and strongly interact with light and other electromagnetic (EM) waves. Because of their intricate helical structure, new properties of CLC have emerged revealing unique optical behavior that has resulted in rewriting Braggs’ law for how light interacts with soft materials. The aim of this work is to push the limits of spectral tuning with a new method of augmenting light-cholesteric interactions using a polymer-sustained conical helix (PSCH) structure. We experimentally explore the reversibility of reflective wavelength modulation and validate the mechanism enhanced by a polymer-sustained helicoidal structure *via* theoretical analyses. The conical helix structure of a CLC, formed by low-field-induced oblique orientation of cholesteric helices, is comprised of a chiral dopant, a conventional nematic, and bimesogenic and trimesogenic nematics. Polymerizing a small amount of a reactive mesogen in the CLC with an applied electric field produces a templated helical polymer network that enables three switched optical states, including light-scattering and transparent states as well as color reflection in response to an applied increasing or decreasing electric field. An electro-activated PSCH optical film covers a wide color space, which is appropriate for tunable color device applications. We envisage that this PSCH material will lead to new avenues for controlling EM waves in imaging and thermal control, smart windows and electronic papers.

## Introduction

The potential applications of optical modulation have stimulated considerable interest in exploring new topological configurations in stimuli-responsive active soft materials with tailored functionalities^1–4^. Cholesteric liquid crystals (CLC) possess a periodic helical structure, and are known for their selective reflection of light which occurs when the wavelength (*λ*) matches the pre-selected pitch (*P*_*o*_) of the helix such that *λ* = *P*_*o*_*cosθ*, where *θ* is the angle between the light propagation and helical axis^5^. Dynamically and precisely controlled pitch modulation in a CLC may be achieved without disturbing the orientation of the helical axis, and thus producing a color-tuning effect^6–9^ that finds applications in mirrorless tunable lasers^10^, thermography^11^ and switchable notch filters^12^. The ability to manipulate the photonic band would be highly desirable, but is challenging due to the strong perturbation of the periodic helical structure in a cholesteric LC in response to an external stimulus such as an electric or magnetic field, as described by Helfrich^13^. The strategies employed to induce pitch modulation include the use of an interdigitated-electrode structure and electric fields to generate wavelength modulation^14^. Polymer stabilization is an alternative method to achieve this goal in which a polymer template is formed along the cholesteric helix avoiding any field-induced breakdown and permits the tunability of the reflective band in a narrow spectral range^3,9,15^. Here, we present a field-induced augmentation of Bragg reflection in a CLC that exhibits a memory effect arising from an interconnected-granular polymer network.

Since the recent discovery of the twist-bend nematic (N_TB_) phase in bimesogens or trimesogens consisting of molecules containing two or three rigid rod-like mesogenic units interconnected with odd-membered flexible chains, there has been a steep rise in the number of studies exploring their structure-property relationships^16–21^. These achiral, flexible and bent twist-bend nematogens exhibit nanoscale helical structures were theoretically predicted several decades ago^22^^,^ and only confirmed experimentally recently^18,23,24^. The self-assembled heliconical structures in N_TB_ phase formed between crystalline and nematic phase, consists of racemic mixture of left- and right-handed helices. These are quite different from heliconical structures in CLCs that are induced with electric field and, due to chiral additive in the mixture, form either left—or right-handed helix. Heliconical cholesteric liquid crystals have wide-ranging application potential in devices such as image sensors^25^, lasers^10^, and light modulators^26^. In general, the oblique helicoidal structure of the CLC generates a reflection band over a broad spectral range with a narrow bandwidth due to the small effective birefringence. The self-assembly of an oblique helicoidal structure occurs only upon reducing the electric field for an homeotropically aligned sample under a high electric field, as experimentally demonstrated by Xiang *et al*.^10,27^. However, the formation of the electrically-modulated conical helices is irreversible due to the high-energy topological configuration. Polymer stabilization is an effective method to reduce the free energy of a CLC in a high-energy topological configuration. Indeed, the templated polymer network helps not only in providing a memory for reorientation, but also enables a fast reversible modulation of the CLC between different helicoidal pitches or orientations which has not been possible previously^4,28–32^. In this work, we demonstrate the electric field induced color tunability in polymer stabilized cholesterics with conical helix structure while increasing the electric field from off-state focal-conic configuration to on-state homeotropic configuration, via intermediate conical helix states that reflect in visible spectrum. Similarly, the LC cell shows a reverse trend of electric field induced transition while decreasing the electric field.

**Figure 1:**
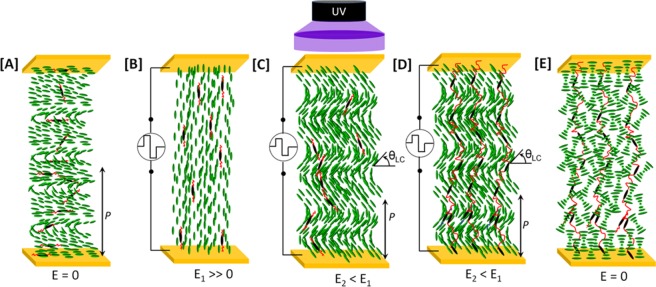
Fabrication of a cholesteric cell with PSCH: (**A**) l CLC with planar alignment in the off-state (E = 0) forming a periodic twisted helical structure. (**B**) Applying a strong electric field (E_1_»0) across the two substrates unwinds the cholesteric helices to form a uniformly-aligned nematic state (**C**) The reactive mesogen in the cholesteric mixture is photopolymerized with an ultraviolet (UV) light source and a conical helix configuration having conical angle θ_LC_ is achieved on lowering the electric field (E_2_ < E_1_) from the uniform nematic phase. (**D**) Formation of polymer-sustained conical helices with the CLC collapsed to form focal-conic domains.

We prepared three mixtures with varied polymer concentrations to systematically investigate the proposed polymer-stabilization approach, namely PS-M1 (1.3% polymer), PS-M2 (1.8% polymer) and PS-M3 (2.5% polymer). For the detailed compositions of the mixtures and the chemical structures of all the components see the *Experimental section*. The test cells filled with CLC mixtures appeared transparent given the configuration of the cholesteric LC and long helical pitch (Fig. 1A). Upon applying a strong electric field along the helical axis, the CLC was unwound and formed a homeotropic nematic phase with the director perpendicular to the substrates (Fig. 1B). The CLC realigned in conical helices with a tilt angle of the director (local orientation of the molecules) between 0° and 90° (Fig. 1C) as the electric field was decreased, and, thus the pitch was varied without disturbing the helical structure. The photopolymerization of the reactive monomer, as pictorially demonstrated in Fig. 1C, is done at 0.6 V/µm at which the cell reflects near red (630 nm). The electric field at photopolymerization is selected such that CLC forms the conical helix at lowest electric field. This ensures the heliconical polymer network having small conical helix angle. The polymer network that mimics the twisted structure of the conical helix (Fig. 1D) was formed by photopolymerization of the reactive mesogen in the mixture under low-intensity UV illumination to minimize the disturbance of the helicoidal texture. After removal of the applied field and returning to the off-state at the end of the photopolymerization, the CLC collapsed to form a weak light scattering texture with randomly-aligned focal conic domains (Fig. 1E) due to the mismatch in helical pitches between the CLC and helix-templated polymer network.

**Figure 2:**
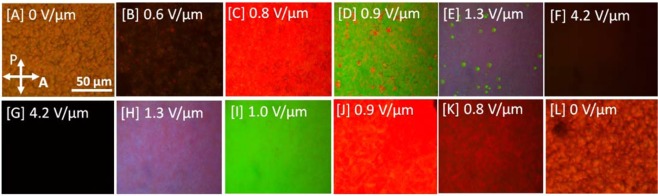
Electrically tunable colors in PS-M1. Polarizing optical microscope images obtained on increasing (**A** to **F**) and subsequently decreasing (**G** to **L**) the electric field showing: focal-conic state (**A** & **G**), conical helix state reflecting blue (**E** and **H**), green (**D** and **I**), and red (**C** and **J**). (**B** and **K**) textures which reflect in the IR region and (**F**&**G**) show the homeotropic state.

Polarized optical microscopy (POM) investigations (For details, see *Experimental section*) revealed that the electrical tunability of the texture for PS-M1 resulted in reversible changes from light scattering to a reflective color and then to a transparent state in response to increasing and decreasing electric field. In reflection mode, viewed between crossed polarizers, PS-M1 shows focal-conic domains in the off-state (Fig. 2A). On gradually increasing the applied electric field, the focal-conic domains begin to form conical helices that reflect light in the near-infrared spectral range at 0.6 V/µm, displaying a dark texture with a deep-red tinge (Fig. 2B). On further increasing the electric field, the test cell selectively reflects red, green, blue and purple colors (Fig. 2C–E). Increasing the electric field to 4.2 V/µm, the cholesteric LC is unwound to form a transparent film with homeotropic configuration and no birefringence, hence displaying a dark texture when viewed through the POM. Reducing the electric field from 4.2 V/µm, the test cell shows a similar sequence of planar textures and reflects blue, green, red, near-infrared and finally, a light scattering texture having focal-conic domains in the off-state (Fig. 2G–L). In terms of voltage, the required amount AC voltage applied to drive the PSCH cell is around 203.7 V. Videos S1 and S2 (Supporting information) show a continuous switching in real time for PS-M1 and PS-M2 respectively from the light scattering texture to reflective colors and to the transparent state. The polymer network formed along the conical helix structure allows for the electrical tunability of the texture, especially while increasing the electric field, and this feature underpins the considerable application potential of this technology in information displays, and electro-optic and photonic devices.

The electric-field-induced deformation of a PSCH can be described by the extended Frank-Oseen free energy equation, *F* = *F*_*el*_ + *F*_*p*_ + *F*_*elec*_, where *F*_*el*_ is the elastic free energy ($$=[\frac{1}{2}{K}_{1}{(\overrightarrow{{\nabla }}\cdot \overrightarrow{n})}^{2}+\frac{1}{2}{K}_{2}{(\overrightarrow{n}\cdot \overrightarrow{{\nabla }}\times \overrightarrow{n}-{q}_{0})}^{2}+$$$$\frac{1}{2}{K}_{3}{(\overrightarrow{n}\times (\overrightarrow{{\nabla }}\times \overrightarrow{n}))}^{2}]$$), *F*_*p*_ is the free energy of the polymer ($$=\frac{1}{2}{W}_{0}si{n}^{2}(\theta -{\theta }_{LC}$$) and *F*_*elec*_ is the electric free energy ($$=\,-\,\frac{1}{2}\Delta \varepsilon \cdot {\varepsilon }_{0}{E}^{2}$$). Thus, the free energy of the system can be expressed by Equation (1):

1 $$\begin{array}{rcl}{\rm{F}} & = & [\frac{1}{2}{K}_{1}{(\overrightarrow{{\nabla }}\times \overrightarrow{n})}^{2}+\frac{1}{2}{K}_{2}{(\overrightarrow{n}\cdot \overrightarrow{{\nabla }}\times \overrightarrow{n}-{q}_{0})}^{2}+\frac{1}{2}{K}_{3}{(\overrightarrow{n}\times (\overrightarrow{{\nabla }}\times \overrightarrow{n}))}^{2}]\\  &  & +\frac{1}{2}{W}_{0}si{n}^{2}({\theta }_{P}-{\theta }_{LC})-\frac{1}{2}{\Delta }\varepsilon \cdot {\varepsilon }_{0}{E}^{2}\end{array}$$

where K_1_, K_2_ and K_3_ are the splay, twist and bend elastic constants, respectively, q_0_ = 2π/p, W_0_ is the anchoring energy along the polymer surface, θ_LC_ is the conical angle of the LC, θ_p_ is the conical angle of the polymer network, ε_0_ is the dielectric permittivity in free space, Δε is the dielectric anisotropy and E is the applied electric field. Owing to the bent molecular shape of the bimesogen and trimesogen, the CLC mixture has K_3_ < K_1_, and this facilitates the formation of the conical helices. A smaller K_3_ value also lowers the bend-to-splay elastic free energy ratio of the system, and that leads to an energetically-favored continuous bend in the LC orientation to form the conical helix or transient planar configuration. On applying a strong electric field, the elastic free energy is dominated by the electric free energy breaking down the helical structure and aligns the LC along the direction of the field. On decreasing the electric field below a threshold field strength, the CLC forms a conical helix with a right-handed rotation of the LC, determined by the handedness of the chiral dopant, and a short P_0_. On further reducing the electric field, the conical angle of the CLC starts to decrease until reaching θ_LC_ (as shown in Fig. 1C). The high-energy conical helix configuration in the CLC with K_3_ < K_2_ exists between the unperturbed helix and the unwound helix within the electric field range that varies between q_0_(K_3_/(ε_0_ΔεK_2_)^1/2^) and q_0_(K_2_/(ε_0_ΔεK_3_)^1/2^)^33^. Due to the weak polar anchoring of the unwound helix, the self-organization of the cholesteric LC in the conical helix configuration is favored upon decreasing the electric field, but the strong planar anchoring of the LC along the rubbed polyimide in the unperturbed helix configuration restricts the formation of a conical helix structure. The polymer stabilization of the conical helices at the lowest conical angle θ_LC*_ provides a memory for the swift reorientation of the helicoidal structure and assists in lowering the energy required to form a conical helix structure, as compared to the system without polymer stabilization in which the molecules would be anchored to the surface in a planar orientation where θ = 0. Thus, on applying the electric field, the PSCH starts to form a uniform helicoidal texture that reflects a red color (Fig. 2C) which changes to green and blue on increasing the field strength, indicating the self-assembly of the conical helix with an increasing cone angle.

**Figure 3:**
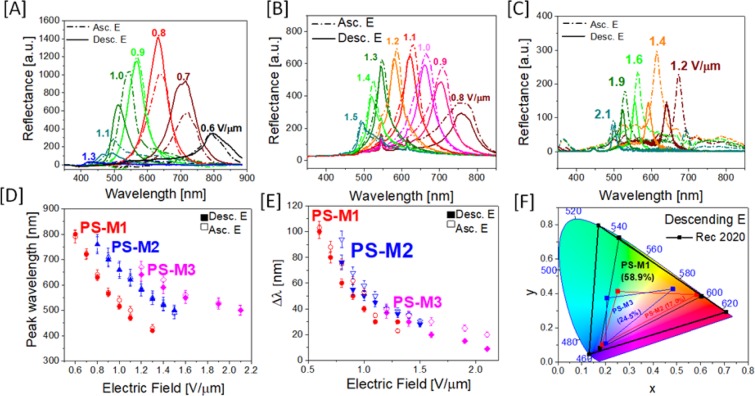
Wavelength-shift in the reflective colors of the three PSCH cells. Typical reflection spectra of PS-M1 (**A**), PS-M2 (**B**) and PS-M3 (**C**) on varying the electric field, (shown for each peak) measured on increasing the (dotted) and decreasing (solid) the applied field. Effect of electric field on peak wavelength (**D**) and bandwidth (**E**) of the reflective peaks. The error bars on the data points represent the standard deviation within 3 measurements on either direction of their mean value represented by the data point. (**F**) Color gamut of PSCH. CIE 1931 chromaticity diagram at 2^0^ viewing angle of reflection wavelength in electrically-switched during decreasing the electric field. The numbers in the plot represents the percentage area covered by the color gamut in comparison to Rec. 2020.

Figure 3 and Video S3 (supporting information) show the dependence of the peak wavelength and bandwidth of the reflection peak on field strength that reveals a wide spectral range of selective reflection from 400 nm to 800 nm. The method used to acquire reflection spectra is discussed in Experimental section. As the field strength is increased, the peak wavelength shifts from red to blue (Fig. 3A–C). This clearly follows the theoretical model described by Meyer that predicts the helical pitch of a cholesteric LC to be inversely proportional to the applied electric field^33^. On decreasing the electric field, the peak wavelength shifts from blue to red. The Bragg reflection follows the equation: mλ = P_0_cosθ, where m is an integer value, λ is the peak wavelength of reflection, and θ is the angle of incident light. Here, it is important to note that the reflectivity at each peak wavelength differs mainly due to two reasons: 1) the effective birefringence (Δn_eff_) is a function of θ:

$${{\rm{\Delta }}n}_{{\rm{eff}}}=\frac{({{\rm{n}}}_{{\rm{e}}}{{\rm{n}}}_{{\rm{o}}})}{\sqrt{{n}_{e}^{2}co{s}^{2}\theta +{n}_{o}^{2}si{n}^{2}\theta \,)}}-{{\rm{n}}}_{{\rm{o}}},$$

where n_o_ and n_e_ are the ordinary and extraordinary refractive indices, and the number of pitches sandwiched within the cell varies with p. Thus, at stronger field strengths, the effective birefringence is smaller which results in a lower reflectivity of blue. Similarly, the reflectivity of red is also low due to the decreased number of cholesteric pitches in the test cell with a fixed thickness. It is also important to consider the scattering effect in a polymer sustained conical helix that increases with polymer concentration and reduces the reflectivity of the cell. PS-M1 having 1.3% polymer shows a maximum peak reflectance, whereas PS-M2 (1.8% polymer) and PS-M3 (2.5% polymer) show a decreased peak reflectance due to high scattering from the grained polymer network. The wide spectral tuning range in PS-M1 (400–800 nm) also gets shorter in PS-M2 (500–800 nm) and PS-M3 (500–700 nm) with the increase in polymer concentration. Interestingly, the electric field required for complete spectral tuning in the PSCH increases with the polymer concentration. Hence, it is necessary to optimize the appropriate polymer concentration to achieve a sustainable polymer network with fast switching dynamics and wide spectral tuning without increasing the electric field required to obtain the spectral tuning. We also found that electric-field-induced reflection from conical helix is extremely sensitive to the composition of the mixture. A minor change in the composition does result in small but identifiable peak shift. Without photopolymerization, we observed a peak shift of ~ 20 nm on slight change in the composition. However, it was also noted that a major change in composition (>10%) of bimesogen or trimesogen content does not result in any conical helix configuration. For all our polymer-stabilized CLC mixtures, we obtained a reversible reflection peak over the entire spectral range on increasing and decreasing the electric field. The peak wavelength and bandwidth (Δλ) of the reflection peaks follows the inverse trend with respect to the applied electric field on increasing and decreasing the electric field (Fig. 3D,E). The electric field driven modulation of the helical pitch in the PSCH has a small hysteresis meaning that the peak wavelengths achieved on increasing and decreasing the electric field are very close. Error bars on the datapoints represent the standard deviation in the peak wavelength or Δλ measured independently for three different samples. A small variation in cell thickness (±0.2 µm) affect the reflection properties of PSCH device at same electric field. Thus, repeatability and consistency of the device properties strongly depend on mixture composition, cell thickness and electric field. Δλ for all the reflection peaks increases with decreasing electric field (Video S3). Δλ depends on the effective birefringence Δn_eff_ and p: Δλ = Δn_eff_ p. Thus, at lower electric fields, both Δn_eff_ and p increase which results in broader reflection peaks at longer wavelengths. We observed that PSCH cell shows no significant change in spectral tunability at low polymer concentration in comparison to PSCH without polymer stabilization. Figure S1 shows the POM and photographic images, and reflection spectra for the PSCH cell before photopolymerization. In contrast to mixtures with polymer network, the CLCs with heliconical structures exhibited spectral tuning only while lowering the electric field from homeotropic configuration to light-scattering state as observed by us while experimentally characterizing the mixtures without polymer or it is extremely slow that it takes about few minutes to switch from light scattering state to homeotropic state, as briefly described by Xiang *et al*.^27^. However, with polymer network, the reflectance from the device reduces significantly due to scattering effect from the polymer network. There was ~20% loss in reflectance with 1.3% polymer concentration as compared to reflectance from the device without polymer network. Inclusion of polymer network also narrows spectral tunability range from 400–800 nm without polymer, to 500–700 nm with 2.5% polymer concentration. All the reflection studies above were performed at normal incidence. However, the reflected wavelength will change if observed at an angle, following the equation of Bragg’s reflection (mλ = P_0_cosθ). The angular dependence of the reflected wavelength in PSCH cells will require another set of studies, but it is evident from the equation that reflected wavelength will have blue shift as observer’s angle goes away from normal incidence.

We have evaluated the color gamut of the reflective colors achieved in polymer-sustained conical helices using the standard called Rec. 2020 used for LCD evaluation. One of the reasons behind using this standard is to quantify our spectral purity using the most stringent and comprehensive color standards applied by many technologists and research groups. Here we compare our reflective colors on CIE 1931 color space which is commonly used in the display industry and provides a simplified method to assess the quality of the device for a wide variety of applications. We define the spectral tuning range in terms of the percentage of coverage area of the color gamut for PSCH in comparison to Rec. 2020. Figure 3F illustrates the color gamut for all three mixtures with the polymer sustained conical helix on increasing and decreasing the electric field. The color gamut region covered by PS-M1 shows a large area coverage that encompasses 58.9% of Rec. 2020 in the CIE 1931 color space on decreasing the field and 46.7% (Figure S2 in supporting information) while increasing the field. In the color space, PS-M1 (1.3% polymer) expands outwards and close to the locus of the color space that represents high purity of blue, green and red color, which is rare in reflective CLCs reported in the literature^3,34^. There is an obvious shrinkage in color gamut of the polymer-sustained conical helices with higher polymer concentrations due to significant light scattering which in turn deteriorates the purity of the reflected colors.

**Figure 4:**
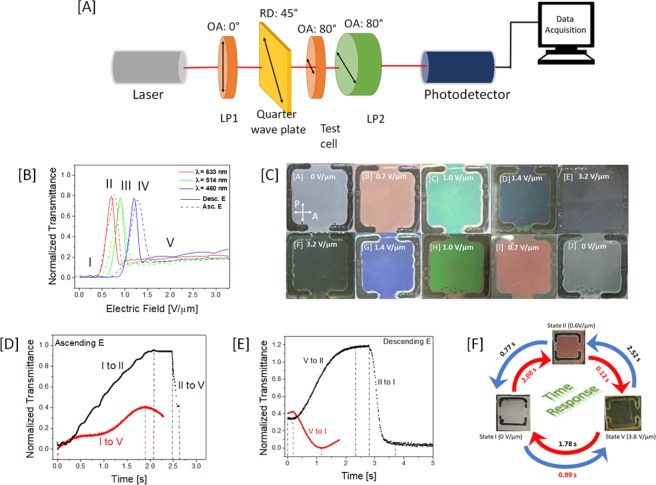
Electro-optics of PS-M1. (**A**) Schematic illustration of optical setup for electro-optical studies of polymer-sustained conical helix. (**B**) Transmittance vs. electric field for PS-M1 on increasing and decreasing the electric field with laser sources having different peak wavelengths. (**C**) The photographs of the cell with different textures on increasing and decreasing the electric field. (**D**) Transmittance vs. time during the switching process from the light scattering state to the transparent state *via* color reflective state. (**E**) Transmittance vs. time during the switching process from transparent state to light scattering state via color reflective state. (**F**) The response time measured in a trimodal switching process between the light scattering and color-reflective *via* the transparent state while increasing and decreasing the electric field. The response time measurements were carried out with a He-Ne laser.

For the application like color smart window, the switching properties of PSCH device need to be studied to ensure that response time of the device is within few seconds. The switching characteristics in PS-M1 have been investigated using an electro-optic setup that includes a linear polarizer (LP1), the PSCH cell, another linear polarizer (LP2), and a quarter wave plate in the same order as listed. Figure 4A shows the schematic illustration of optical setup used for electro-optical studies. Both linear polarizers are crossed at 80°. The optical axis (OA) of quarter wave plate is aligned along OA of LP2 at 80°. The rubbing direction of test cell is aligned at 45° to LP1. The electro-optics of the PSCH cell was studied using differing probe light sources, specifically, blue (460 nm), green (514 nm) and red (633 nm) lasers. Other details on electro-optic study are mentioned in *Experimental section*. The driving electric field required to achieve homeotropic configuration in a PSCH cell is 3.6 V/µm. It was observed that the transmittance-electric field curves measured during increasing (dotted lines) and decreasing electric field (solid lines) do not completely overlap, which explains the hysteresis in transmittance that arises due to low polymer concentration (Fig. 4B). This effect is re-emphasized in the photographic cell images (Fig. 4C) captured in different textures on increasing and decreasing electric field, where the test cell shows a visible difference in its optical texture observed at the same electric field while ramping the field strength up or down. The cell images in Fig. 4C are captured while ramping up the electric field approx. at the rate of 0.05 V/µm every 5 s. Initially in the off-state, the cell is gradually switched to transparent state via color-reflective state, and then switched back to light-scattering state by gradually reducing the applied electric field. We observed that cell shows much brighter color with wider bandwidth while ramping up the electric field, as compared to ramping down. This is possibly occurring during high anchoring energy of the LCs along the polymer network that tend keep some domains of focal-conic texture intact even when other domains have already switched to conical helix configuration. This anchoring energy distribution does show an effect on spectral purity difference while ramping up and down. At a low electric field, the test cell shows a light scattering texture (State I) that blocks all the light, giving low transmittance. On directly switching to 0.6 V/µm, the test cell forms a conical helix that reflects red light (State II) which interacts with the incident polarized light (λ = 633 nm) to transmit only left circular polarization while reflecting the right circular polarization. However, the incident light does not interact with the conical helices reflecting green (State III) or blue (State IV). As the test cell switches to the transparent state at 3.6 V/µm (State V), the polarized light from LP1 without any phase retardation, but some light leakage across LP2 as they are not completely crossed (80°), and a small transmittance is measured by the photodiode detector. The transmittance-electric field curves measured with green (λ = 514 nm) and blue lasers (λ = 460 nm) shows distinct peaks at green and blue reflecting textures, indicating transmission of left circularly polarized light at different electric fields. It is important to note that the transmittance of left circularly polarized light passing through a quarter wave plate for 3 different lasers looks identical due to normalization. The transmittance-time curves were measured for switching between states I, II and V in PS-M1 (Fig. 4D and E). The response times between different states in PS-M1 are around 2.5 s (Fig. 4F) which makes the polymer-sustained conical helices very relevant for smart window applications. Interestingly, the response time of switching from a light scattering to a transparent state via red reflective state (2.2 s) is much longer than direct switching from a light scattering to a transparent state (1.8 s). The same occurs when switching the other way round from a transparent to a light scattering state. This reflects the slow dynamics of PS-M1 when self-assembling the cholesteric in the form of a conical helix due to low polymer concentration that partially templates the helix configuration. The switching process from a red reflective state and a light scattering to a transparent state is fast due to the strong applied field strengths. The overall switching cycle of the PSCH from a light scattering state to a transparent one and vice versa on increasing and decreasing the electric field, respectively, is completed in less than 5 s. We achieved the response time of switching from light-scattering state to transparent state via color-reflective state in device with polymer network (few seconds) that is much faster than without polymer network (2 minutes), as reported in Xiang *et al*.^27^.

**Figure 5:**
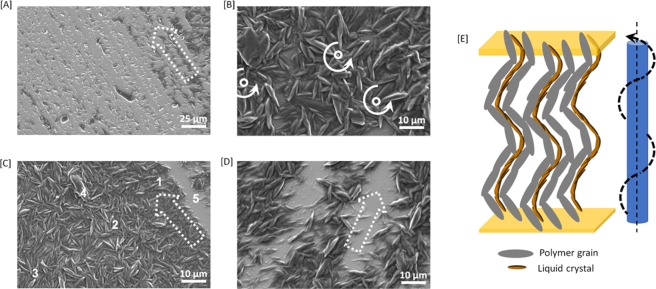
Polymer morphology in PS-M1: SEM images of the granular polymer network mimicking the conical helix pattern on the top (**A**–**C**) and bottom substrates (**D**) of the test cell. The white dotted arrows indicate the rubbing direction of the polyimide layer. The circles and circular arrows represent the helical axis and orientational rotation of polymer grains, respectively, as observed from the top. (**E**) Schematic representation of the granular polymer network formed along the conical helix. The figure is not to the scale as the length of polymer granules is 2000 times that of length of the liquid crystal molecules.

The morphology of the polymer network generated by the photopolymerization-induced phase separation in PS-M1, was investigated using scanning electron microscopy (SEM). The equipment details and method used for sample preparation is discussed in *Experimental section*. The continuous polymer walls, composed of interconnected granular particles, are anchored along the rubbing direction (represented by the white dotted arrow) on the top (Fig. 5A,B,C) and bottom glass substrate (Fig. 5D). While the granular polymer network is denser in some regions (Fig. 5B), there are some regions with scarce but uniformly distributed walls (Fig. 5A). The flexible spacers in RM257 maintain the orientation along the LC, despite the disruption of nematic order during photopolymerization, thus forming anisotropic (rice-grain-like) interconnected and continuous aggregations. A typical rice-grain in the aggregates observed on the top and bottom substrates is 8–10 µm in length and 1–2 µm in diameter. To avoid the formation of a dense fibrous polymer network that can significantly increase the driving electric field required to generate color-tunability, we carried out the photopolymerization without any photoinitiators. In the absence of photoinitiators, the rate of the photopolymerization reaction (R_X_) is significantly slower which leads to a reaction-limited aggregation process dominating, especially for a low concentration of reactive mesogen. The aggregates of the reactive mesogen, formed due to strong interactions along the aromatic rigid cores, are excited by absorbing 365 nm UV light and can abstract a hydrogen to undergo a self-initiation process, as described by Weng *et al*.^35,36^. This can possibly lead to the formation of a continuous granular morphology, rather than a fine-fibrous network. As modelled by Rajaram *et al*.^37^, the radius of the granular particles (*ξ*) in the continuous network is related to the viscosity of the medium *η*, Boltzmann constant *K*_B_, temperature *T* and rate of reaction *R*_*x*_ by Equation (2):

2 $$\xi \sim {(\frac{KT}{\eta {R}_{x}})}^{\frac{1}{3}}({\phi }^{2/9})$$

Considering the low rate of reaction *R*_*x*_ in the initiator-less photopolymerization and low viscosity of the medium, a thick polymer morphology (large ξ) would be formed, as observed in Fig. 5(A–D). On exposing the mesogenic monomers to UV light at low electric field where the CLC reflects in the red, the polymerized granular network structure allows efficient stabilization of the conical helix (Fig. 5E). The polymer grains near the top substrate (region 1) observed in Fig. 5C, are much smaller in diameter and length as compared to those at the bottom substrate (Fig. 5D). The marked gradient in the rate of reaction across the thickness of the cell due to the gradient in the intensity of UV light (strong on the top substrate nearest to the UV source and weakest at the bottom substrate), results in a fine-granular morphology at the top substrate and a bulky-granular morphology on the bottom substrate. In some regions on the substrate nearest to UV light, the layer thickness of the densely packed granular network is much higher (region 2) due to the collapsed polymer network from the other substrate during the mechanical separation of the two glass substrates. The corresponding domains on the bottom substrate with no polymer can be observed in Fig. 5D. Figure 5C shows the morphology of the polymer network inter-connecting the substrates and reveals a gradient beginning with dense packing in the bulk (region 3) with an aggregation process dominated by a reaction-limited process. The propagation of dislocations along the plane of the surface creates significant defect lines during the self-organization of a conical helix^27^, some of them can be observed in the POM images (Fig. 2), leading to a localization of the reactive mesogens along the defects that create a densely packed granular polymer network in the bulk. The large chunks of aggregates (region 4) with granular network are observed in the SEM image. The empty domains formed along the rubbing direction of the substrate (region 5) indicate that some regions have a more uniform orientation of conical helices with fewer non-uniformities in granular network (Fig. 5A), while others have more non-uniformities (Fig. 5B–D). These non-uniformities in the granular structure could be the result of non-uniform photopolymerization rate of reactive monomer across the cell. The discrepancy in size distribution, location and orientation may arise from one or a combination of the following factors; the rubbing defects of the liquid crystal alignment, the electric-field-induced tilt angle of the helicoidal structure of the CLC, gradient light-cholesteric helices interactions *via* Bragg reflection, and inhomogeneous light energy distribution from a non-collimated UV light source. The anisotropic polymer network in the bulk has a strong aligning effect on the LC which can be explained using Equation (3) that provides a mathematical relationship between polymer concentration (c) and effective aligning field (E_p_)^38^.

3 $${E}_{p}={(\frac{{\pi }^{3}K}{(8+{\pi }^{2}){\varepsilon }_{0}|\Delta \varepsilon |})}^{1/2}\frac{\sqrt{c}}{R}$$

where K is the average of the splay and bend elastic constants, ε_0_ is the permittivity of free space, R is the radius of the polymer grains. When K~4.8 pN (K_1_~2.5pN, K_3_~7pN), Δε~6.5, c = 0.013 (1.3%) and R = 1 µm, the value of *Ep* for PS-M1 is ~0.1 V/µm. However, we observed that additional electric field of 0.2–0.4 V/µm is required to achieve the conical helix that reflects at same wavelength after photopolymerization in PS-M1. Although an underestimated value is predicted by equation 3 for electric field required to form conical helix that reflects at same wavelength after polymer network formation, equation 3 provides a first-order estimate to calculate the amount the extra power required to operate the device. From equation 3 it can be very well predicted that increase in the polymer concentration will certainly increase the electric field required to demonstrate wide-spectral tuning. The intrinsic nature of a gradient distribution of a polymer in this PSCH composite although increasing the level of challenges in fabricating devices with a narrow reflection bandwidth, does provide opportunities for applications that will require a wide-reflection band.

In conclusion, we have explicitly demonstrated an electric-field augmented Bragg reflection tuning with a polymer-sustained conic helix of a cholesteric liquid crystal. Apart from the electrically-tunable selective reflection of light, the electro-active composite exhibits two other optical states namely, the focal conic that blocks light, and the homeotropic that is transparent. Theoretical analyses validate the formation of a grain-shaped interconnected polymer network and its alignment effect as well as the responses of different states of PSCH structures to external electric fields. The memory effect produced by a granular polymer network not only mirrors the conical helix, but also facilitates a long-range pitch modulation in a cholesteric while increasing and decreasing the electric field. The dynamics of a switching process between a color-reflective and transparent state of a conical helix is substantially-enhanced from almost unobservable switching to a few seconds with polymer stabilization while ramping up the electric field from light-scattering state to transparent state, via color-reflective state. A wide color gamut with high spectral purity and uniform structural colors across the entire electrode area is achieved with the PSCH. We envisage that this work will open new avenues for applications in the burgeoning field of smart windows, home decor, color e-writers and energy-harvesting applications.

## Experimental section

### PSCH mixtures

The CLC mixtures used in the preparation of polymer-sustained conical helix (PSCH) films comprised of a calamitic nematic LC (5CB), a bimesogenic LC (CB7CB), a trimesogenic LC (CB6OBO6CB), a chiral dopant (R5011) and a reactive mesogen (RM257). The chemical structures of all the constituents are:

**Table:** 

**Material**	**Chemical Structure**
5CB	
R5011	
CB7CB	
CB6OBO6CB	
RM257	

The composition of constituents in the mixture is tabulated below:

**Table:** 

Mixture	5CB (wt.%)	CB7CB (wt.%)	CB6OBO6CB (wt.%)	R5011 (wt.%)	RM257 (wt.%)
PS-M1	49.7	43.3	4.8	0.8	1.3
PS-M2	50.0	42.8	4.8	0.8	1.8
PS-M3	51.0	41.1	4.6	0.8	2.5

### Cells

The CLC mixture was filled between two glass substrates separated by 50 µm glass spacers via capillary action to achieve a uniform gap (d) of 48.5 ± 0.2 µm. The inner surface of the glass substrates was coated with a conductive indium-tin-oxide (ITO) layer and a layer of rubbed polyimide PI2555 (Nissan Chemicals) to enable homogeneous alignment of the CLCs at the surface with a preferred direction. The CLC mixtures were filled into the electro-optical (EO) test cells (assembly of two glass substrates) at an elevated temperature in the isotropic phase (90 °C).

### Polarized optical microscopy (POM) images

To examine the tunable LC configurations in PS-M1, the test cell was observed under a polarizing optical microscope (POM). All the POM images were taken with Nikon Optiphot2-pol at room temperature. The POM images of PS-M1 (Fig. 2A–L) were captured using 10X objective with settling time of 1 s to ensure a uniform texture across the exposed region in the test cell. The illumination of the light source with constant intensity was maintained while capturing all the POM images.

### Reflection spectra

The reflection spectra of PS-M1, PS-M2, PS-M3 were measured using spectrophotometer (USB2000+, Ocean Optics) with tungsten-halogen light source (LS-1, Ocean Optics) having beam diameter of 0.4 mm and responsive wavelength range of 360–825 nm. All the measurements were carried out at room temperature. All the reported measurements are as obtained from spectrophotometer without any data processing, such as normalization.

### Electro-optical Characterization

The PSCH cells were placed between two linear polarizers that are crossed at 80°. A He-Ne laser source (λ = 633 nm) is used as a probing light, while an Ar^+^ laser is used with color filters for monochromatic light with λ = 514 nm and 460 nm. The transmitted light intensity is measured using a photodiode as a function of electric field from 0 to 3.2 V/µm. To measure the switching time between the scattering (0 V/µm), reflecting (0.6 V/µm) and transparent (1.6 V/µm) states, the transmitted intensity was measured as a function of time during the switch-on and switch-off processes and the characteristic response time was defined as the time taken to reach the transmittance of the respective state. All measurements were performed at room temperature.

### Scanning electron microscopy

The glass substrates of the cells containing the PSCH templated network were carefully disassembled after immersing in liquid nitrogen to ensure minimum damage to the structure of the polymer network. Gold nanoparticles were sputtered on the surface of the polymer network to increase conductivity. An FEI Quanta 450 scanning electron microscope was used for morphological investigations.

## Supplementary information

Supplementary information
