# Supplementary material for: Augmenting Bragg Reflection with Polymer-sustained Conical Helix
Source: Sci Rep. 2019 Apr 2;9:5468. doi: 10.1038/s41598-019-41836-4 (PMC6445128; doi:10.1038/s41598-019-41836-4)
Supplement: Supplementary file 1 — Supplementary information [file 41598_2019_41836_MOESM1_ESM.docx]

Augmenting Bragg Reflection with Polymer-sustained Conical Helix

*Vinay Joshi^*^, Daniel A. Paterson, John M.D. Storey, Corrie T. Imrie, Liang-Chy Chien^*^*

Vinay Joshi and Liang -Chy Chien

Chemical Physics Interdisciplinary Program and Liquid Crystal Institute, Kent State University, Kent 44242, USA.

Daniel A. Paterson, John M.D. Storey, Corrie T. Imrie

Department of Chemistry, School of Natural and Computing Sciences, University of Aberdeen, Aberdeen AB24 3UE, Scotland UK.

*Correspondence to: vjoshi2@kent.edu; lchien@kent.edu.

**Supporting Information**

A self-assembled conical helix is produced in a cholesteric LC mixture that has a smaller value of the bend elastic constant (K_3_), as compared to the twist elastic constant (K_2_). Recently, there has been extensive research on structure-property relationships in bimesogenic and trimesogenic liquid crystals (LC) that comprise of two or three rigid rod-like mesogenic units, respectively, separated by odd-membered flexible spacers that exhibit the twist-bend nematic (N_TB_) phase. Molecular modelling has revealed that on average they exist in bent molecular conformations and consequently exhibit low K_3_ values, and this has been confirmed experimentally.^2–4^ With the motivation to achieve a conical helix structure that can interact with ambient light to generate Braggs’ reflections encompassing the photonic bandgap, we prepared cholesteric LC mixtures consisting of a conventional nematic LC: 5CB, chiral dopant: R5011, bimesogen: CB7CB, trimesogen: CB6OBO6CB and reactive mesogen: RM257. Prior to the photopolymerization of the reactive mesogen in the cholesteric mixture, we investigated the field-induced color-tunability in PS-M1. To produce an electrically tunable selective reflection of light, the cholesteric LC is unwound by applying a strong electric field and subsequently lowering the field to form conical helices with an oblique orientation of LC director (0˚<θ_LC_<90˚). Figure S2A shows the POM images of PS-M1 on decreasing the electric field from the unwound transparent state to various conical helix states with color reflection from blue-green-red and a light-scattering focal-conic state at zero field. The photographic images (Figure S2B) of the test cell show the uniformity of the texture over the entire electrode area. Figure S2 also presents the reflection spectra, peak wavelength and bandwidth at various electric field that demonstrate the broad range of controlled reflectance that covers the entire visible range. The range of electric fields to achieve color-tunability is slightly lower before photopolymerization [0.4-0.9 V/µm] than after [0.6-1.3 V/µm] which reflects the aligning effect of polymer network.


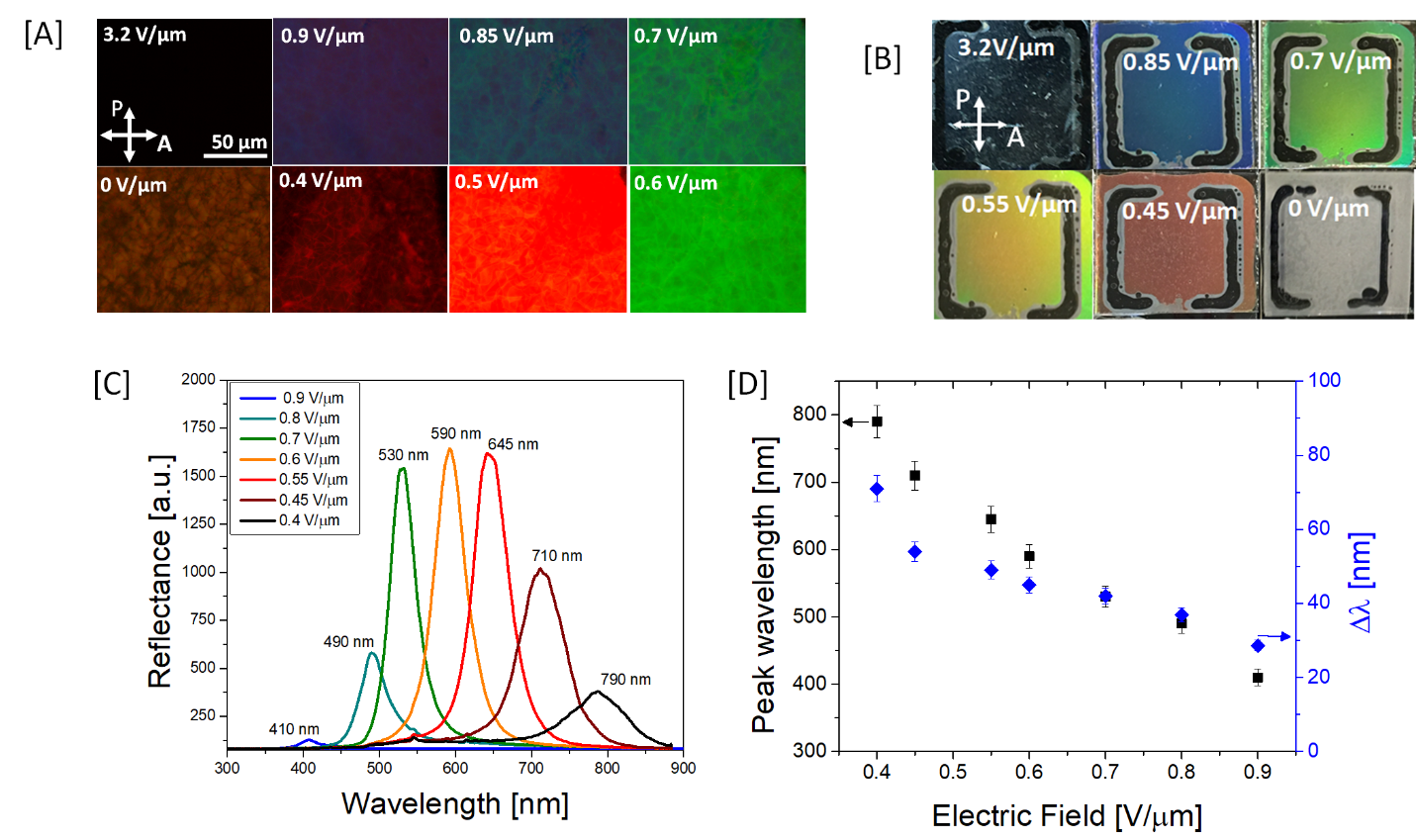


Figure S1: PS-M1 before photopolymerization: POM images [A] and photographic images [B] while decreasing the electric field. [C] Reflection spectra while reducing the electric field. Peak wavelength and bandwidth (Δλ) as a function of electric field.

Figure S2: Color gamut of PSCH. CIE 1931 chromaticity diagram at 2⁰ viewing angle of reflection wavelength in electrically-switched during increasing the electric field. The numbers in the plot represents the percentage area covered by the color gamut in comparison to Rec. 2020.
